# Decoupling Magnetic and Electric Field Control in Magneto-Ionic Materials for Energy-Efficient Brain-Inspired Memory Devices

**DOI:** 10.1021/acsami.5c19791

**Published:** 2025-12-24

**Authors:** Luis Martínez Armesto, Zheng Ma, Huan Tan, Eva Pellicer, Irena Spasojevic, Jordi Sort

**Affiliations:** † Departament de Física, 16719 Universitat Autònoma de Barcelona, Bellaterra (Cerdanyola Del Vallès) 08193, Spain; ‡ Institució Catalana de Recerca i Estudis Avançats (ICREA), Pg. Lluís Companys 23, Barcelona 08010, Spain; § Catalan Institute of Nanoscience and Nanotechnology (ICN2), CSIC and BIST, 08193 Barcelona, Spain

**Keywords:** energy efficiency, nitrogen magneto-ionics, exchange interactions, magnetization modulation, synaptic-like functionalities

## Abstract

Magneto-ionic materials, which enable nonvolatile control of magnetism through voltage-driven ion migration, are emerging as promising candidates for neuromorphic computing. Unlike conventional memristors, these systems allow dual actuation by both electric and magnetic fields, providing a broader range of functional capabilities. The reliance on voltage rather than current significantly reduces Joule heating and enhances the energy efficiency. However, the general need for external magnetic fields to modulate the voltage-induced magnetic response remains a key limitation, undermining the full energy-saving potential of these systems. In this work, we present a magneto-ionic strategy in CoFeN that fully decouples the electric and magnetic field requirements. By taking advantage of a planar N^3–^ ion migration and the ferromagnetic exchange interactions between preexisting and newly generated CoFe magnetic regions, we achieve remanent-state magnetization control solely through applied voltage. The system exhibits behaviors reminiscent of neuromorphic-inspired functionalities, such as synaptic potentiation and depression, while also exhibiting a cumulative voltage-driven increase in magnetization in the absence of a magnetic field. Once the magnetic field is switched off, synaptic weight remains influenced by both the sample’s magnetic and electric history. By eliminating the need for magnetic fields, our approach contributes to reduce energy consumption, offering a more efficient pathway for brain-inspired magneto-ionic devices.

## Graphical abstract

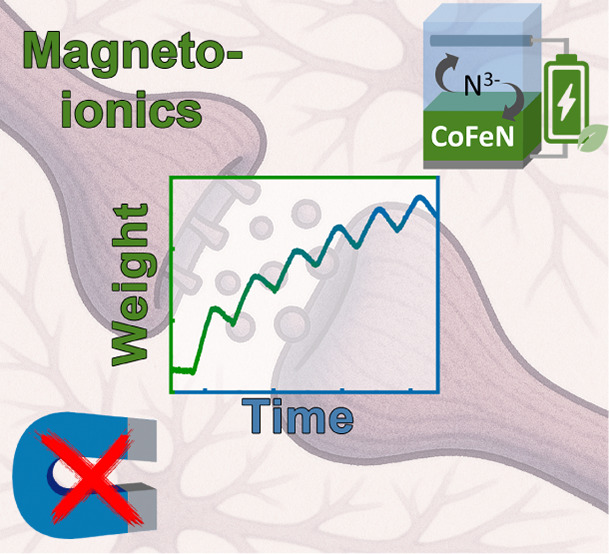

## Introduction

In recent years, advancements in Artificial Intelligence (AI), along with the increasing adoption of the Internet of Things (IoT) and Big Data, have increased the societal demand for integrating these emerging technologies into everyday applications. However, the energy consumption associated with these innovations is escalating exponentially. A fundamental factor contributing to this trend is that state-of-the-art computing systems rely heavily on electric currents for data processing and storage. As a result, the Joule effect causes these devices to generate significant heat, leading to considerable energy loss during computation. This inefficiency is worsened by the additional power required for thermal management and cooling systems.^1,2^ To overcome these limitations, researchers are increasingly exploring alternative computing paradigms that can reduce energy consumption while maintaining high performance.

Currently, AI technologies are predominantly based on software neural networks, which depend on extensive data sets and still utilize conventional computing paradigms, where memory and processor are separate subunits (i.e., von Neumann architecture). The continuous transfer of data between the memory and the processor over a communication bus consumes a lot of time and energy resources. An alternative approach involves the implementation of nonvon Neumann architectures to construct artificial neural networks in hardware, where neurons (and synapses) may be able to simultaneously compute and store information (i.e., in-memory computing), mimicking the way the human brain works.^3,4^ To achieve this, a device capable of replicating neuronal functions and synaptic interactions would be highly desirable. These emerging technologies frequently rely on memristors, which exhibit neuromorphic characteristics such as synaptic plasticity, specifically potentiation and depression, that depend both on the amplitude and time of electric stimuli.^5,6^ Despite their improved efficiency compared to software-based neural networks, these resistive-based hardware implementations remain reliant on electrical currents, inherently sustaining Joule heating. Thus, novel materials and designs are needed to reduce energy losses associated with resistive switching while enabling reliable and scalable neuromorphic hardware implementations.

In recent years, magneto-ionic materials have gained significant attention because they enable nonvolatile control of magnetic properties via voltage-induced ion migration.^7,8^ The most commonly studied ionic species include H^+^ ^9,10^ , Li^+^,^11–13^ O^2–^,^14–16^ and, more recently, N^3–^.^17–20^ CoN has been reported to offer not only faster response times than its oxide counterpart (CoO_*x*_), but also ion propagation through a planar migration front,^17^ facilitating improved control and efficiency. The ternary compound (Co–Fe–N) has been shown to outperform binary Co–N in terms of magnitude and speed of magnetization changes, and cyclability (i.e., magneto-ionic endurance).^19^ Remarkably, magneto-ionic materials can emulate important synaptic behaviors, including potentiation, depression, or learning under deep-sleep conditions.^21,22^ Also magneto-ionics has been found useful for reservoir computing.^7,22–24^

In most magneto-ionic studies to date, the observation of voltage-induced magnetic changes typically requires the application of both electric and magnetic fields. While an electric field can modulate the amount of ferromagnetic phase generated through ion migration, external magnetic fields are utilized to orient and influence the properties of such a magnetic phase. Interestingly, recent research reveals that combining magnetic fields with voltage allows precise tuning of synaptic depression linearity, mirroring neuromodulation in biological systems and enhancing learning accuracy.^25^

In this work, we present a novel approach to manipulate the magnetic response of CoFe, magneto-ionically formed from paramagnetic CoFeN. We first utilize the simultaneous application of voltage and magnetic field to modulate the amount of generated CoFe and its orientation. Unlike previous magneto-ionic cycling studies that required sustained magnetic fields, we show that, after the magnetic field is removed, the system retains magneto-ionic cyclability, emulating synaptic potentiation/relaxation cycles driven exclusively by an electric field. By working in magnetic remanence and avoiding the use of an external magnetic field during magneto-ionic cycles, the energy consumption is reduced by several orders of magnitude (10^6^ for an applied field of 10 kOe) compared to the state-of-the-art in magneto-ionics.

The amplitude of the magnetization cycles (i.e., weight changes) depends on the sample’s prior magnetic and electric history. We find that this behavior arises from a planar N^3–^ ion migration in CoFeN that drives a layer-by-layer growth of ferromagnetic CoFe within the paramagnetic matrix, with exchange interactions enforcing parallel alignment between the magnetization of the newly formed and preexisting CoFe sublayers. By fully decoupling voltage application from the external magnetic field, this method has the potential to enhance the energy efficiency of magneto-ionic materials while minimizing heat losses from the electromagnets employed to generate the fields.

## Experimental Section

### Sample Preparation

50 nm-thick Co_0.35_Fe_0.65_N (for simplicity, CoFeN) ternary nitride films were grown at room temperature by magnetron cosputtering on [100]-oriented Si substrates, previously coated with 20 nm of Ti (adhesion layer) and 50 nm of Pt that serves as the bottom electrode (see Figure 1a,b). An AJA International ATC 2400 sputtering system with a base pressure of around 1 × 10^‑7^ Torr was used. The Si/Ti­(20 nm)/Pt­(50 nm) substrates were partially masked during the nitride deposition to allow electrically contacting Pt afterward, leaving an exposed area of 5 × 5 mm^2^. The target-to-substrate distance was 11 cm. Magnetron sputtering was performed at a total pressure of 3 mTorr under an Ar atmosphere for metallic depositions (Ti and Pt) and a mixture of Ar and N_2_ for the Co_0.35_Fe_0.65_N ternary nitride layer. The Ar:N_2_ flow ratio was set to 1:1 in order to provide the nitrogen-rich atmosphere needed for the growth of the paramagnetic nitride film. To obtain the Co-to-Fe ratio corresponding to the most magneto-ionically active state (i.e., Co_0.35_Fe_0.65_N^19,26^ ), a metallic Fe target was connected to a 50 W DC power supply, while the Co target was powered by a 55 W RF source.

**Figure 1:**
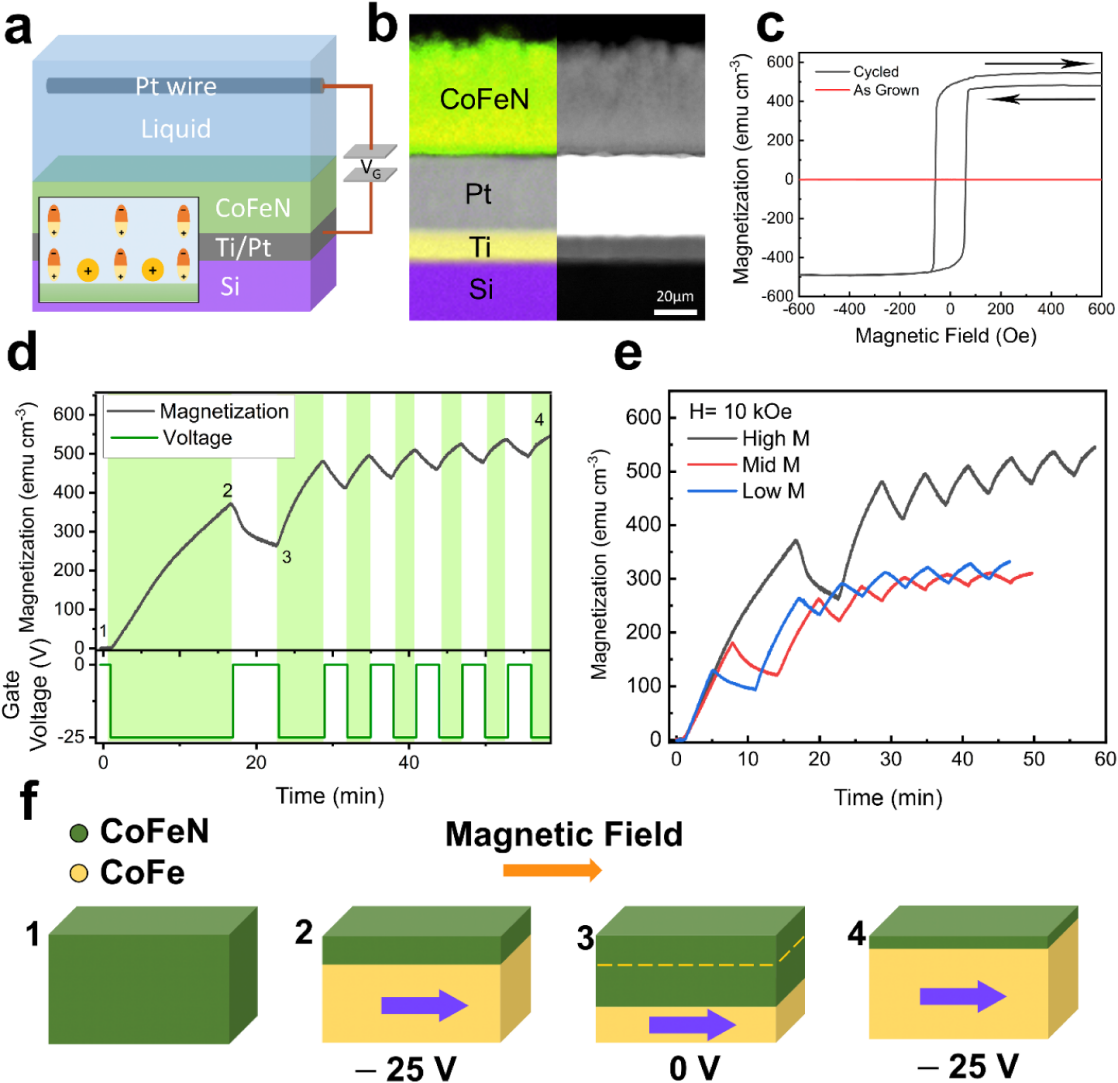
a) Schematic representation of the magneto-ionic cell, Na^+^ ion (in orange) accumulation and formation of the electric double layer (inset) under the application of a gate voltage (*V*_G_). b) Electron microscopy images of the multilayer stack in the as-grown state (left: EELS elemental mapping; right: HAADF-STEM). c) Hysteresis loops of the as-grown sample (in red) and after the voltage treatment indicated in (d), point 4) (in black). d) Magnetization vs time curve (top), under the action of gate voltage pulses (bottom), highlighting the periods where voltage is applied using a green background. This measurement is performed by superimposing an external magnetic field *H* = 10 kOe. e) Magnetization vs time cyclability curves obtained after three different initial conditioning states, switching off the gate voltage (−25 V) after 16, 8, and 4 min, respectively, that correspond to the black, red, and blue lines. These states are designated as “High M”, “Mid M”, and “Low M”, respectively. f) Schematic representation of the CoFeN ↔ CoFe transformations (at points 1–4 from panel (d)) when magnetic and electric fields are applied. The orange arrow represents the direction of the applied magnetic field, while the violet arrow indicates the magnetization direction in CoFe. Note that, for simplicity, in the schematic diagrams, we refer to the magneto-ionically generated ferromagnetic layer as “CoFe” although, as evidenced by HRTEM, grains of metallic Fe and Co, as well as N-depleted ferromagnetic phases, are also identified.

### Magnetoelectric Measurements

Magnetoelectric measurements were performed in a vibrating sample magnetometer (VSM) from Micro Sense LOT-Quantum Design at room temperature while gating the films using a liquid electrolyte consisting of propylene carbonate (PC) with Na^+^ and OH^–^ ions in solution. Gating voltages were applied between the Pt bottom electrode and the Pt wire in a capacitor-like configuration^27,28^ using an external Agilent B2902A power supply. Magnetic fields were applied along the in-plane direction. Two primary measurements were conducted: (i) Magnetic moment vs applied magnetic field hysteresis loops, from which the linear contribution arising from nonmagnetic sources, such as the sample holder or substrate, was subtracted, and (ii) Magnetic moment vs time curves under the influence of two different types of physical stimuli: electric fields and magnetic fields. Electric fields were generated through the formation of an electric double layer (EDL) in the liquid electrolyte when a voltage was applied. When negative voltage is applied, the dipolar nature of PC causes the molecules to orient with the positive end of the dipole facing downward toward the material’s surface, while Na^+^ ions also migrate toward the material/electrolyte interface, forming an EDL. The EDL is a dielectric layer of less than 1 nm in thickness that enables a strong electric field (several MV cm^–1^) using moderate voltage.^27,29,30^ Solvated ion species (Na^+^, OH^–^) reinforce the ionic strength of the EDL that forms upon voltage application. The sample was initially subjected to in-plane magnetic fields, which were subsequently turned off to investigate magneto-ionic effects driven solely by voltage. Although the magneto-ionic response for CoFeN can occur on the ms scale,^31^ we performed minute-scale cycling on purpose to enable a clearer comparison of the response with and without an applied magnetic field. Previously reported approaches to increase ion migration speed, namely those operating at the material level (e.g., reducing the thickness of the magnetoelectric material, optimizing its conductivity, or introducing defects) or at the electrolyte level (increasing the electrolyte’s ionic strength)^31–34^ were not attempted here. Some of these approaches rely on modifying the microstructure of the magneto-ionic target to enhance ion migration, but such changes might adversely affect the exchange interactions.

### Structural/Compositional Characterization

To examine the structural and compositional properties of the samples, high-angle annular dark-field scanning transmission electron microscopy (HAADF-STEM), high-resolution transmission electron microscopy (HRTEM) and electron energy-loss spectroscopy (EELS) analyses were conducted using a Spectra 300 STEM microscope operated at 200 kV located at the Joint Electron Microscopy Center at ALBA Synchrotron. Cross-sectional thin lamellae were prepared by focused ion beam milling after the deposition of an electrically conducting Pt–C on top of the heterostructures to enhance electrical conductivity (i.e., minimizing charging effects) as well as to prevent them from oxidation and contamination from ion beam damage. The lamellae were subsequently transferred to a Cu TEM grid.

## Results and Discussion

### Magneto-Ionic Generation of CoFe from CoFeN: Simultaneous Action of Electric and Magnetic Fields

Figure 1a shows a schematic of a homemade electrolytic cell. The CoFeN film is placed inside a polymeric cell. The Ti/Pt layers serve as the bottom electrode, while a Pt wire was used as the counter electrode, placed approximately at 3 mm from the sample. Anhydrous PC with dissolved (i.e., solvated) Na^+^ (10–25 mg L^–1^) and OH^–^ ions was used as the liquid electrolyte. Na^+^ and OH^–^ arise from the reaction between water and metallic sodium, which was previously introduced in PC to eliminate the trace amounts of water in the electrolyte.^17^

Figure 1b shows the HAADF-STEM image of the Ti (20 nm)/Pt (50 nm)/FeCoN (50 nm) stack together with the corresponding EELS compositional mapping of all the layers. As shown in Figure 1c, in its initial state, the as-grown CoFeN sample is fully paramagnetic, whereby Fe, Co, and N are homogeneously distributed across the CoFeN film (Figure S1). To extract N^3–^ ions from the sample into the liquid electrolyte, a −25 V gate voltage difference is applied between the bottom Pt electrode and the Pt wire counter electrode. Removal of N^3–^ ions from the CoFeN induces the formation of a ferromagnetic CoFe layer, typically at the bottom of the magneto-ionic target material for sufficiently high magneto-ionic rates.^19,26^

In this first experiment, a 10 kOe magnetic field is continuously applied during negative voltage gating in order to fully align the magneto-ionically generated CoFe layer (from points 1 to 2 in Figure 1d). When the gate voltage is set to 0 V, N^3–^ redistribution takes place in the system, resulting in the partial reformation of CoFeN, leading to a reduction of the saturation magnetization (*M*_S_) with time (relaxation), as shown in Figure 1d,e and illustrated in Figure 1f (from points 2 to 3). The relaxation process is also evident from the hysteresis loop of the voltage-treated sample (Figure 1c and Figure S2), measured after the voltage cycling (point 4), which shows an opening between the descending and the ascending branches of the loop, indicating a continuous decrease of *M*. To confirm that the origin of this opening is temporary, a second hysteresis loop was measured, and a negligible opening was then obtained (Figure S2). After the first relaxation, magneto-ionic cyclability was investigated by applying a series of −25 V/0 V pulses with a periodicity of a few min (points 3 to 4). Figure 1d shows that after each cycle, the overall moment increases. This behavior is reminiscent of synaptic plasticity,^22^ where after each “learning” and “forgetting” iteration the system shows a clear net potentiation effect.

Additionally, the capability to hold and operate in different memory states was explored. The overall *M* increase when applying −25 V depends on the duration of the first gate voltage segment. By tuning its duration between 4, 8, and 16 min, three different states were defined, which were designated as “Low”, “Mid”, and “High” moment states (Figure 1e). These different states can be interpreted as being analogous to different learning stages, where the connection between two neurons would be softer or stronger. Inspection of the cycles for the different states in Figure 1e reveals that, for lower learning times, the relaxation (or forgetting) is slower. On the other hand, the “High *M*” state shows higher-amplitude cycles, which means that both potentiation and relaxation are more intense than in the “Low *M*” state, and the overall *M* increase after several cycles is also clearly higher. This behavior emulates time-dependent synapse weight changes (i.e., neuromorphic plasticity). Namely, in the biological brain, it is easier to retain a larger portion of the information learned in 16 min than in 4 min. Note, however, that the Mid *M* sample does not strictly follow this trend. The peculiar behavior of this sample will be further discussed in subsequent sections.

### Magnetization Modulation in the Absence of Magnetic Field

Next, the magnetic state recovery and the magneto-ionic cyclability are investigated in the absence of a magnetic field. To estimate the efficiency of the remanent-state magneto-ionic process, one could calculate the ratio between the energy required to apply a magnetic field of 10 kOe using our VSM in a continuous manner with that needed to actuate the film magneto-ionically at remanence. As energy equals power (voltage × current) multiplied by time, the ratio can be obtained from the respective power values. In our case, this corresponds to (104 V × 6.5 A)/(25 V × 10^–5^ A) = 2.7 × 10^6^. Hence, the energy consumed in the presence of the magnetic field is 10^6^ times higher than when only an electric field is applied, underscoring the benefit of the magnetic field decoupling strategy. Note that this estimate does not include the additional energy required to refrigerate the electromagnets, which would further increase the total energy cost in the presence of the magnetic field.

First, the system is conditioned using simultaneous electric and magnetic stimuli as in the previous section, applying −25 V for 16 min (curve from points 1 to 2 in Figure 2a). Then, after the system is relaxed for 3 min (from points 2 to 3), the external magnetic field is switched off and cyclability is performed solely under the action of voltage, at *H* = 0 Oe (see transition from black to red curve in Figure 2a). When the magnetic field is removed, the ferromagnetic layer is left in a remanent state (*M*_R_), which causes an abrupt decrease in *M* (i.e., drop from magnetic saturation to remanence). Subsequently, *V*_G_ = −25 V is applied for 6 min (from points 4 to 5), causing an increase of *M*_R_ due to the regeneration of the CoFe ferromagnetic layer. Since no external magnetic field is present, the spins of the newly formed CoFe layer align via exchange coupling (*J*) with the underneath CoFe magnetic layer (see schematic drawings in Figure 2c). Remarkably, *M*_R_ after cyclability (point 7) is higher than *M*_S_ when a magnetic field was still applied (point 2), evidencing the effectiveness of magnetic exchange interactions to retain the orientation of the CoFe film. Even in the remanent state, synaptic-like behavior is maintained.

**Figure 2:**
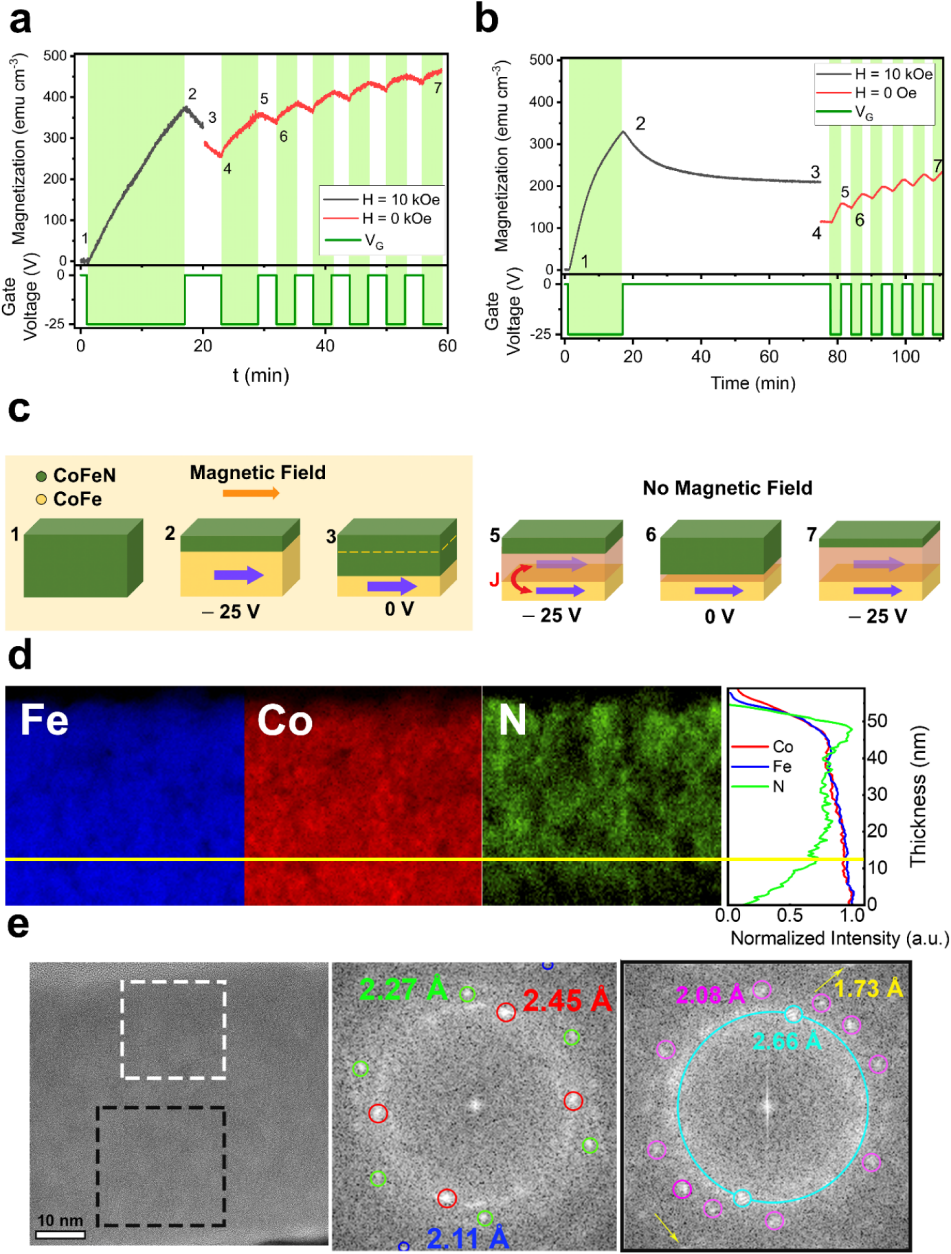
a,b) Magnetization vs time curves. The black line represents the magnetization when *H* = 10 kOe is applied and the red line when *H* = 0 Oe. The bottom panels indicate the dependence of the gate voltage vs time. Green shadowed areas are drawn to highlight the time intervals in which gate voltage *V*_G_ = −25 V is applied. c) Schematic representation of the CoFeN and CoFe dynamic transformations when magnetic and/or electric fields are applied, at different stages of the cyclability process (as indicated with numbers in panels a) and b)) (“High *M*” sample). The orange arrow represents the direction of the magnetic field. The violet arrow represents the magnetization of the CoFe, being slightly transparent when no magnetic field is applied. The translucid yellow represents the magneto-ionically generated CoFe layer aligned via exchange interactions with the bottommost CoFe. d) EELS mappings of Fe (blue), Co (red) and N (green) in a CoFeN lamella after applying −25 V for 16 min with a relaxation time of 1 h (state 3 in c)). Shown on the right is the EELS intensity normalized for each element as a function of thickness. The yellow line is a visual guide to correlate the N depletion both in the elemental mappings and the intensity profiles. e) HRTEM image of the same CoFeN lamellae and Fourier Transforms of regions at the topmost part (white dashed square) and at the bottom (black dashed square).

The effects of the relaxation time (from points 2 to 3 in Figure 2a,b) on cyclability were also investigated. In Figure 2b, the same procedure was followed, but the system was left under 0 V for 1 h instead of 3 min before removing the magnetic field. In this case, the amplitude of the cycles is smaller, which means that, either the magneto-ionics dynamics after long-term relaxation is slower or the exchange interactions are less effective in retaining the original orientation (probably because of the formation of a multidomain structure in CoFe over time). Note that for both long-term and short-term relaxation, *M*_R_ (point 7) overpasses *M*_S_ after relaxing (point 3).

To shed further light on the magneto-ionic mechanism, we prepared a lamella from the cross-section of a sample treated with −25 V for 16 min and relaxed for 1 h while applying *H* = 10 kOe (point 3 in Figure 2b). As can be inferred from the EELS elemental mappings in Figure 2d, the nitrogen appears to be mostly depleted from the bottom of the layer, while Co and Fe remain homogeneously distributed (see the elemental profiles at the panel on the right in Figure 2d). To investigate the generated crystalline phases, HRTEM images were acquired, and the crystalline planes were resolved. Fast Fourier transforms of the topmost and bottommost regions were analyzed to determine the interplanar distances. As shown in Figure 2e, three different distances appear at the topmost part: 2.45 Å, which coincides with the (111) plane of (Co,Fe)N (*F4̅3m*), 2.27 Å, which matches the (020) plane of Co_2_N (*Pnnm*), and 2.11 Å, which coincides with the (200) plane of (Co,Fe)­N (*F4̅3m*) or the (111) plane of FCC Fe (*Fm3̅m*). Since the topmost part of the layer contains a significant fraction of N and the initial state of the sample is nonmagnetic, Fe is discarded, thus leaving the (Co,Fe)N and Co_2_N phases as the most plausible options in that region. In the bottommost part of the treated film, different interplanar distances can be observed: 2.66 Å, which matches the (020) plane of Fe_2_N (*Pbcn*), the (100) plane of the CoFe alloy (*Pm-3m*) and the (200) plane of Fe_5_Co_3_ (*Im3̅m*). An interplanar distance of 2.08 Å corresponds to the (111) planes of the Fe and Co FCC phases (*F4̅3m*), while the 1.73 Å spacing matches the (220) plane of Fe_2_N (*Pbcn*) and the (200) plane of Co FCC (*F4̅3m*). All of these phases are ferromagnetic, supporting the interpretation shown in Figure 2c, where the nitrogen depletion at the bottom and accumulation at the top of the films are consistent with the proposed planar magneto-ionic mechanism.

### Tunable Long-Term Multistate Memory States

We also investigated the system’s ability to retain distinct long-term memory states using protocols analogous to those described in the previous section. To this end, we examined the cyclability performance of the “High *M*”, “Mid *M*”, and “Low *M*” samples. Each sample was first actuated with −25 V for different times depending on the desired final state (16, 8, and 4 min, respectively) and then allowed to relax for 3 min under a magnetic field *H* = 10 kOe, after which the field was set to *H* = 0 Oe (Figure 3a). A behavior similar to that observed under magnetic field conditions (Figure 1e) is noticeable with samples exhibiting higher cycle amplitudes correlating with higher initial magnetic moments. Remarkably, after each voltage cycle, the samples exhibit a net increase of magnetization even in the absence of an external magnetic field, evidencing effective exchange interactions between preexisting and newly generated CoFe counterparts at remanence. This behavior is evident even for the “Low *M*” sample. Figure 3b shows the system response after a relaxation period of 1 h, followed by the removal of the magnetic field. In this case, the final *M*_R_ values of the “Low *M*” and “High *M*” samples exceeded their respective *M*_S_ values after the relaxation stage (before *H* = 0 Oe). However, for the “Mid *M*” sample, the *M*_R_ was lower than that of the “Low *M*” sample; the cycles exhibited smaller amplitudes, and the overall recovery remained below the final saturation level.

**Figure 3:**
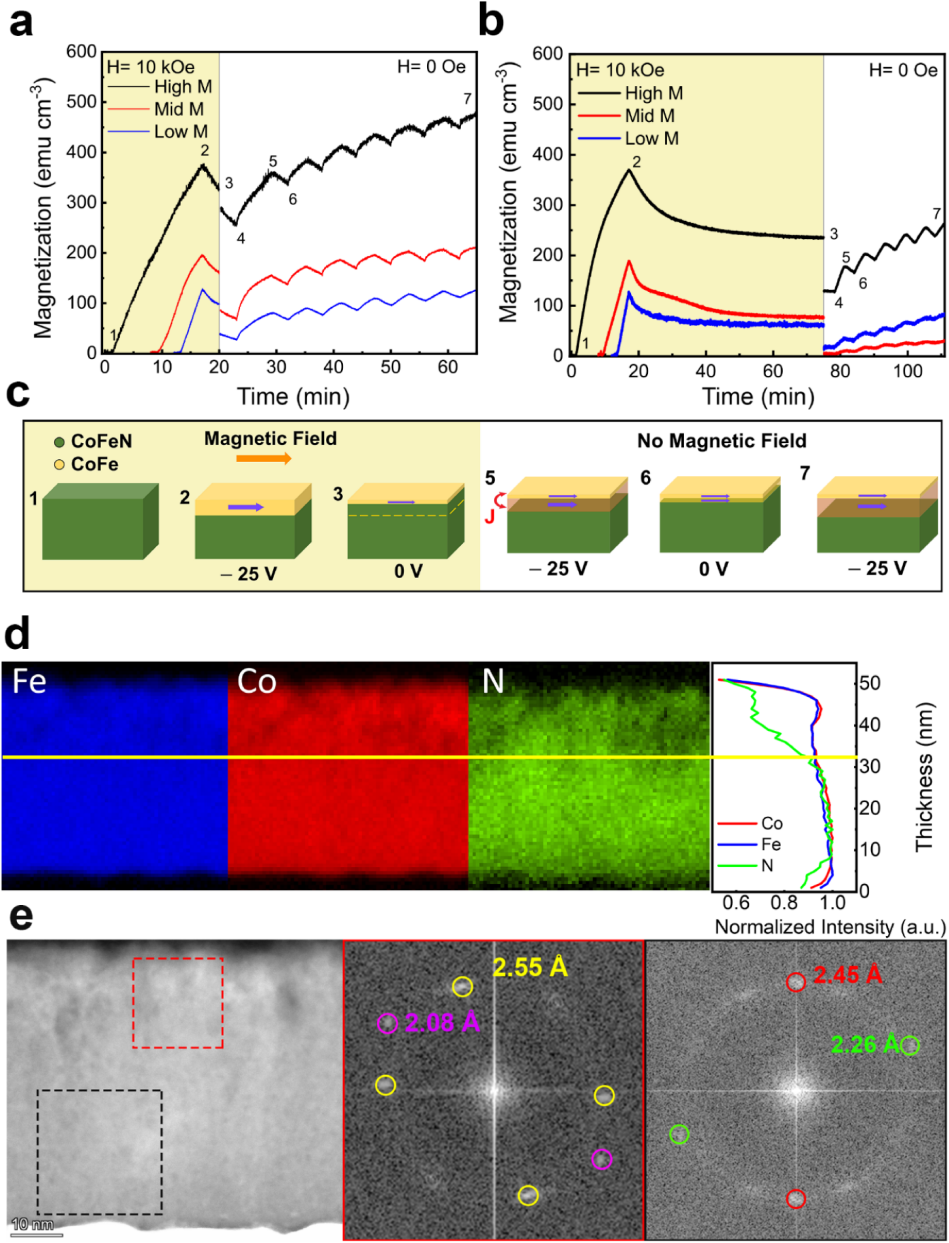
a,b) Magnetization vs time curves of three different states investigated: High, Mid, and Low moment. The yellow background highlights the time interval where *H* was set to 10 kOe. Mid and Low moment curves were shifted in the time axis for the sake of clarity to match the times at which the field is switched off. c) Schematic representation of the dynamic transformations between CoFeN and CoFe when magnetic and/or electric fields are applied, at different stages of the cyclability process (as indicated with numbers in panels a) and b)) for the “Low M” sample. d) EELS element mappings of Co, Fe, and N of a CoFeN lamella after applying −25 V for 4 min (“Low *M*” state), step 3 in panel c). Shown in the panel on the right is the EELS intensity normalized for each element as a function of thickness. The yellow line is a visual guide to correlate the N depletion both in the elemental mappings and the intensity profiles. e) HRTEM image of a CoFeN lamella and the Fourier Transforms of a region at the topmost part (red dashed square) and at the bottom (black dashed square).

Figure 3d shows the EELS elemental mappings of CoFeN lamellae treated by applying −25 V for 4 min followed by a relaxation of 1 h under *H* = 10 kOe (corresponding to the “Low *M*” sample, before cycling). While it is subtle, the planar front in the nitrogen map remains discernible. However, the nitrogen distribution is reversed, i.e., nitrogen depletion occurs at the top and accumulation at the bottom of the film. In fact, in nitrogen-based magneto-ionic systems, nitrogen ions are initially released from the upper parts of the films into the electrolyte in a layer-by-layer manner, leading to nitrogen depletion near the film–electrolyte interface. This behavior is typically observed when magneto-ionic effects are relatively low, or, as in this case, during the early stages of the magneto-ionic process. However, as more nitrogen is released, the local solubility limit of nitrogen in PC can eventually be exceeded. Once this limit is reached (e.g., after fast magneto-ionics or long-term voltage application), further nitrogen release is hindered, causing nitrogen ions to accumulate near the film surface.^19,26^ As a result, nitrogen depletion then occurs deeper within the film, particularly near the substrate interface, as was observed in the “High *M*” shown in Figure 2d.

Figure 3e shows representative HRTEM images and the Fourier transforms of the top part (red) and the bottom part (black) of the “Low *M*” treated film with a relaxation time of 1 h. The top part shows two sets of planes with an interplanar distance of 2.55 Å, which matches the (110) planes of the Fe_4_N (*Pm-3m*) phase (ferromagnetic), together with diffraction spots at 2.08 Å, which match the (111) Fe and Co (*F4̅3m*) FCC phases (ferromagnetic). The bottom parts of the films show spots at 2.45 and 2.26 Å, which match the (111) plane of (Co,Fe)­N (*Fm3̅m*) and (020) Co_2_N (*Pnnm*), respectively. This suggests that the phases present in the bottom parts of the “Low *M*” sample are similar to those in the upper parts of the “High *M*” films (Figure 2e), as expected since both contain larger concentrations of nitrogen.

The behavior described above suggests a complex dynamic in nitrogen ion migration. In the early stages (as seen in the “Low *M*” sample), nitrogen is primarily removed from the topmost region of the film, leaving the bottom layers richer in nitrogen. As the process progresses, however, nitrogen begins to deplete from the bottom and accumulates near the surface (as observed in the “High *M*” sample). This reversal occurs when the rate of nitrogen release at the surface becomes slower than the rate of nitrogen migration within the film. This shift in migration dynamics appears to be reflected in the magnetization versus time (*M vs t*) plots. All three samples initially exhibit a similar *M vs t* slope, but after approximately 10 min, the slope for the “High *M*” sample decreases. Specifically, the “High *M*” sample shows a steeper magnetization increase before the 10 min mark, followed by a more gradual slope afterward, suggesting a transition in ion migration behavior at that point. Interestingly, this transition time aligns closely with the voltage actuation duration in the “Mid *M*” sample, where the gate voltage (*V*_G_) is applied for 8 min. If nitrogen is transitioning from the bottom to the top of the film when the cycling begins, the absence of a well-defined planar migration front could reduce the effectiveness of exchange interactions between preexisting and newly formed ferromagnetic regions. The proposed mechanism is illustrated in Figure S3. When the “Mid M” sample is subjected to 0/–25 V cycles with and without a magnetic field, there is an interplay of two effects: (1) a new FM FeCo sublayer is generated at the bottom due to N^3–^ migration toward the top of the sample, and (2) consequently, the top part transitions from FM to paramagnetic, thus losing moment. As this “magnetic trade” does not occur for the Low M and High M samples, the magnetization increase upon cycling is higher in both cases than for the Mid M. Additionally, if the bottom ferromagnetic CoFe planar front is yet not well defined (i.e., as expected in the early stages of the gating), the exchange interactions at remanence will not be as efficient as for the other two cases. This may result in a slower magneto-ionic response and could account for the complex behavior observed in Figures 1e and 3b for the “Mid *M*” sample.

### Efficiency of Magnetic Exchange Interactions in Modulating Synaptic Weight

In this section, we quantify the efficiency of magnetic exchange interactions in modulating the synaptic weight in the absence of a magnetic field. This is done by comparing the changes in magnetization (expressed as the normalized moment per initial nominal volume of CoFeN) during the −25 V/0 V voltage cycles under both *H* = 10 kOe and *H* = 0 Oe. Representative values of saturation and remanent magnetization (*M*_S_ and *M*_R_, respectively) are indicated in Figure S4. We focus on the changes in *M*_S_ (and *M*_R_) between the final point after full cyclability testing (a total of 5.5 cycles) and the minimum observed after the first relaxation cycle, with and without a magnetic field (see Table 1). Since the applied magnetic field (*H* = 10 kOe) is strong enough to fully saturate all ferromagnetic phases present, the increase in *M*_S_ reported in Table 1 directly reflects the amount of ferromagnetic material generated magneto-ionically between points 3 and 4 in Figure 1d (since it is all fully aligned with *H*). In the “High *M*” sample, *M*_S_ increases by a factor close to 2 between the first and last cycles, indicating that the fraction of ferromagnetic phases (primarily CoFe) roughly doubles. In contrast, the “Mid *M*” and “Low *M*” samples show a larger relative increase (by a factor of approximately 3.6), suggesting more extensive ferromagnetic phase formation during cycling.

**Table 1:** Variation of Saturation Magnetization and Remanence during Cycling^a^

	ΔM (emu cm^–3^)			ΔM_R_/ΔM_S_ (%)	
	Δ*M*_S_ 3 min relaxation	Δ*M*_R_ 3 min relaxation	Δ*M*_R_ 1 h relaxation	3 min	1 h
Low M	241.00	98.10	67.81	40.7	28.1
Mid M	189.88	139.65	25.94	73.5	13.7
High M	285.31	215.52	137.41	75.5	48.2

a Δ*M*_S_ refers to the increment of saturation magnetization between points 3 and 4 in Figure 1d. Analogously, Δ*M*_R_ refers to the increment of remanent magnetization between points 4 and 7 in Figure 3a,b.

This enhanced fraction of ferromagnetic phases in “Mid *M*” and “Low *M*” samples is likely due to the absence of a nitrogen-rich upper surface layer (which forms in the “High *M*” sample when the solubility limit of N^3–^ ions in PC is exceeded) that slows down further magneto-ionic activity. It should be noted, though, that the first phases that are generated during magneto-ionic treatments are probably low-moment phases (like Fe_4_N) which have a lower total magnetization than other metallic phases observed in the “High *M*” state (such as FCC Fe, Co, or CoFe). As the actuation proceeds, the material evolves from semiconducting, nitrogen-rich ferromagnetic phases with a high N/metal ratio toward metallic CoFe phases with higher M_S_.^35^ This transformation produces (i) an increase in *M*_S_, because low-moment or even paramagnetic regions convert into higher-moment metallic phases, and (ii) a reduction in ionic mobility, since the emerging metallic layers are known to screen the applied electric field and slow N^3–^ extraction.^32^ In this framework, the larger absolute Δ*M*_S_ obtained in the High M sample is consistent with the dominance of metallic ferromagnetic phases, while the comparatively higher relative Δ*M*_S_ observed in the Mid M and Low M samples arises from their initially faster ionic response combined with the early formation of lower-moment phases.

When the magnetic field is removed, the observed changes in *M*_R_ under applied voltage are attributed to exchange interactions between preexisting and newly formed ferromagnetic regions. Without these interactions, the net magnetic moments in the newly created ferromagnetic grains would remain randomly oriented and the overall magnetization would not increase, even after repeated voltage pulses. Table 1 reveals two notable trends: (i) higher *M*_R_ increments are observed in samples relaxed for 3 min (Figures 3a and S3b) compared to those relaxed for 1 h (Figures 3b and S3c), and (ii) the “High *M*” sample consistently shows larger Δ*M*_R_ than the “Low *M*” sample, regardless of the relaxation time. The Δ*M*_R_/Δ*M*_S_ ratio serves as an indicator of how effectively exchange interactions preserve the magnetic alignment of newly formed ferromagnetic phases. A ratio of 1 implies perfect alignment (100% exchange efficiency), meaning that all newly generated magnetic moments align with the original ferromagnetic direction. After 3 min of relaxation, the Δ*M*_R_/Δ*M*_S_ percentage ranges between 75.5% and 40.7%, whereas after 1 h, it drops to between 48.2% and 13.7%. This decrease suggests that longer relaxation leads to more disordered spins and weaker exchange coupling upon cycling, possibly because the planar front is partially distorted during the recovery process. Moreover, the “Low *M*” sample exhibits Δ*M*_R_/Δ*M*_S_ percentages lower than those of the “High *M*” sample. This may be due to its more nitridated phases, which contain a higher density of disordered or weakly coupled spins. As a result, these phases are less effective at aligning the spins in the newly generated ferromagnetic regions, making them more susceptible to domain formation and producing a smaller *M*_R_ increase relative to *M*_S_. The “Mid *M*” sample exhibits a low alignment (13.6%) after 1 h of relaxation. This may be explained by the fact that, during relaxation, any developing planar front of nitrogen redistribution tends to diffuse or smear out. As this sample represents a boundary case between forming a nitrogen-enriched top sublayer (as in the “High *M*” sample) and a nitrogen-enriched bottom sublayer (as in the “Low *M*” sample), the nitrogen migration becomes frustrated or incomplete. This could result in the formation of discontinuous ferromagnetic regions or clusters with weak exchange coupling, thereby reducing the overall magnetic alignment.

Regarding N^3–^ dynamics under open-circuit conditions (i.e., at 0 V following the application of a negative voltage), we hypothesize that the EDL established during the negative bias is transiently reversed at 0 V to neutralize the accumulated charges, observed as the change in the sign of the measured current (see Figure S5). Thus, during the first seconds at 0 V, N^3–^ ions are reintroduced into the film, as evidenced by the initial fast drop in *M*. Once the EDL has fully restructured at 0 V, the reduction in *M* is governed exclusively by the remaining N^3–^ concentration imbalance in the film, which establishes a chemical potential gradient that drives the diffusion of N^3–^ through the film. This explains why the higher the magnetization, the larger the *M* reduction at 0 V. The FM phases might evolve either to a lower *M* FM phase or directly to a paramagnetic phase. Further experiments or simulations should be performed to elucidate these dynamics.

Remarkably, in spite of having *M* alignment % that do not surpass 76%, in some cases (e.g., the “High *M*” sample with 3 min relaxation) *M*_R_ after cycling is larger than *M*_S_ at the maximum of the first cycle. This clearly supports the idea that, in this system, a net learning capability can be induced even in the absence of a magnetic field, solely under the action of voltage.

## Conclusions

In conclusion, this work disentangles the two principal actuation parameters in magneto-ionic devices, electric field and magnetic field, which had hitherto been applied simultaneously to orient induced ferromagnetic layers. By decoupling these stimuli, we demonstrate more precise and independent control of magneto-ionic processes. Importantly, eliminating the need for external magnetic fields (typically generated by energy-cost-effective electromagnets) enhances the overall energy efficiency of the approach. Crucially, we show that magneto-ionic transformations can be fully driven under zero-field conditions, where the orientation of the newly formed ferromagnetic regions is governed solely by exchange interactions, an approach not previously demonstrated in the literature. This advancement addresses fundamental limitations of earlier studies and opens pathways for versatile tuning of magnetic properties with potential relevance for brain-inspired memory concepts.

Notably, in some cases the voltage-driven magneto-ionic response without magnetic fields exceeds previously established magnetic saturation levels, underscoring the promise of this strategy for nonvolatile, neuromorphic-inspired learning. The ability to reproduce synaptic-like behaviors under zero-field conditions further highlights the unique advantages of exchange-mediated magneto-ionics. Finally, our findings shed light on the complex dynamic behavior of nitrogen ion migration in CoFeN films. The observed change from nitrogen depletion at the topmost part of the actuated films (“Low *M*” sample) to nitrogen depletion at the bottom part of the films (“High *M*” sample) reveals intricate, depth-dependent ion transport mechanisms that merit further investigation. A deeper understanding of these dynamics will be essential for the future design and optimization of magneto-ionic systems.

## Supplementary Material



## Notes

Decoupling magnetic and electric field control in magneto-ionic materials for energy-efficient brain-inspired memory devices which includes 5 supporting figures and authors information (PDF)
