# Supplementary material for: Decoupling Magnetic and Electric Field Control in Magneto-Ionic Materials for Energy-Efficient Brain-Inspired Memory Devices
Source: ACS Appl Mater Interfaces. 2025 Dec 24;18(1):1985–94. doi: 10.1021/acsami.5c19791 (PMC12781102; doi:10.1021/acsami.5c19791)
Supplement: Supplementary file 1 [file am5c19791_si_001.pdf]

Supporting Information

Decoupling magnetic and electric field control in magneto-ionic materials for energy-efficient brain-inspired memory devices

Luis Martínez Armesto, Zheng Ma, Huan Tan, Eva Pellicer, Irena Spasojevic*, Jordi Sort**

L. M. Armesto, Z. Ma, H. Tan, E. Pellicer, I. Spasojevic, J. Sort

Departament de Física, Universitat Autònoma de Barcelona, 08193 Bellaterra (Cerdanyola del Vallès), Spain, E-mail: irena.Spasojevic@uab.cat; eva.pellicer@uab.cat; jordi.sort@uab.cat

J. Sort

Catalan Institute of Nanoscience and Nanotechnology (ICN2), CSIC and BIST, 08193 Barcelona, Spain

J. Sort

Institució Catalana de Recerca i Estudis Avançats (ICREA), Pg. Lluís Companys 23, 08010 Barcelona, Spain

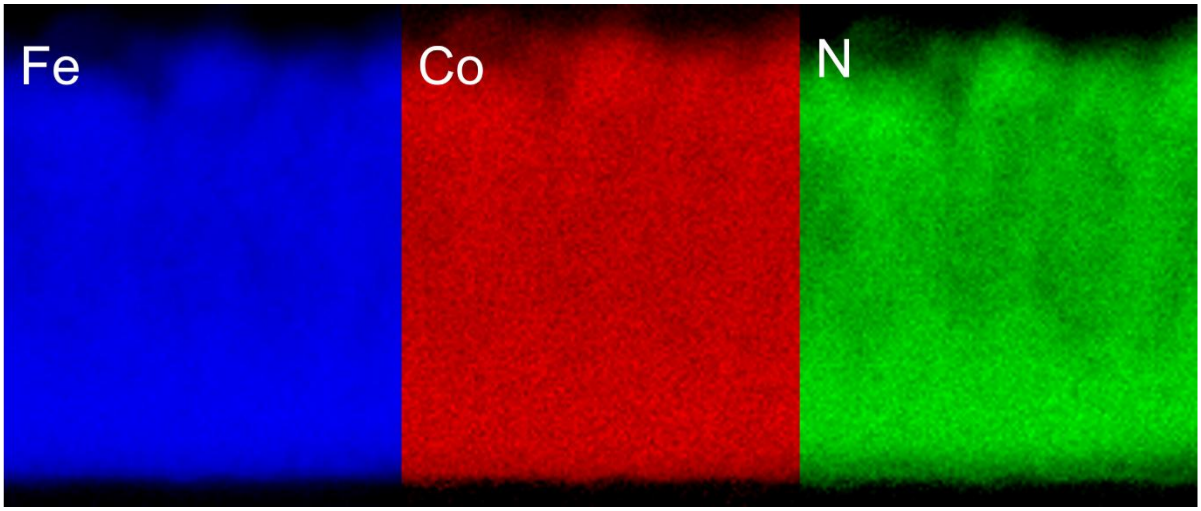

Figure S1. EELS element mappings of Co, Fe, and N of a CoFeN lamellae in the as-grown state.

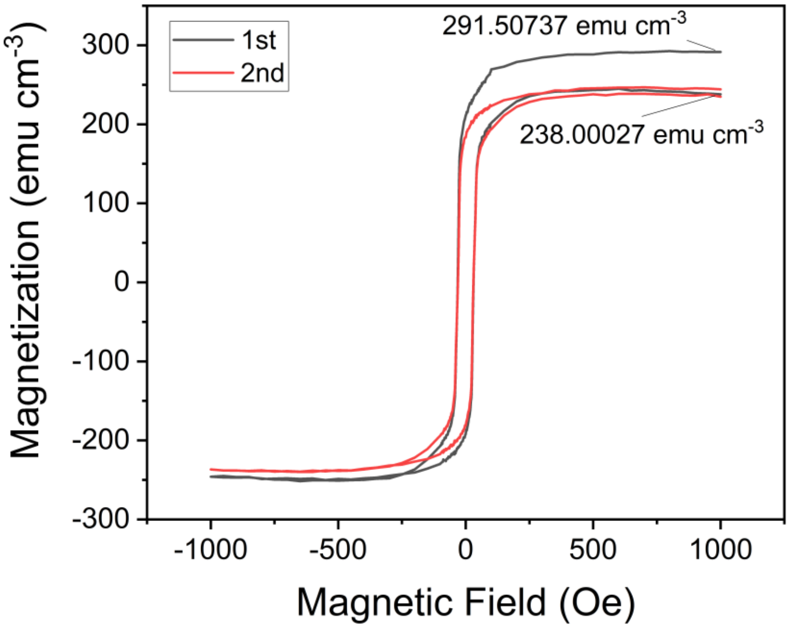

Figure S2. Two hysteresis loops measured after the cycling voltage treatment (with magnetic field applied) indicated in Figure 1(d): 1st loop (black line) just after the voltage treatment, 2nd loop (red line) after 45 min from the voltage treatment.

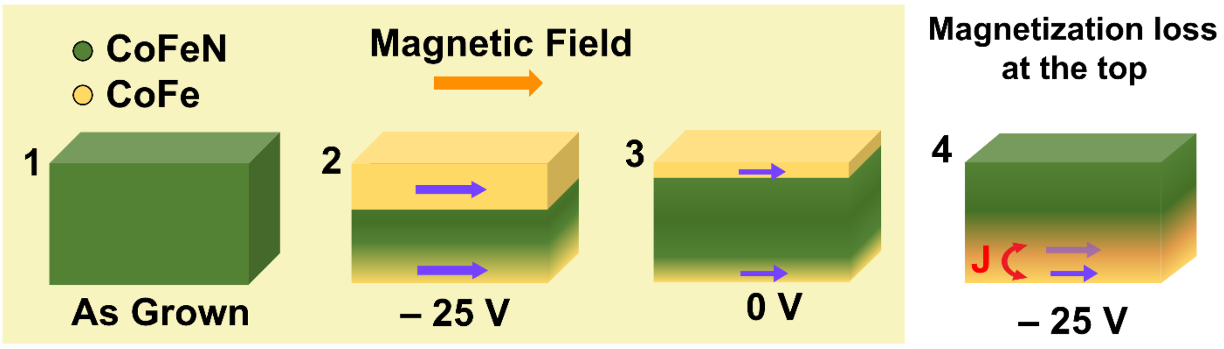

Figure S3. Schematic representation of the proposed N^{3-} dynamics in Mid M sample

Starting from the as-grown sample (1), the nitrogen migration front induced by the combined electric and magnetic field produces a sharp transition from CoFeN to CoFe at the top of the film, akin to Low M dynamics. In contrast, the bottom region develops a more gradual compositional change, characteristic of High M sample, resulting in the formation of two magnetic layers (2). After the application of a negative voltage, open circuit conditions are established (3), and the thickness of both layers relaxes toward their thermodynamically stable state. We propose that in the initial stage, N^{3-} ions are primarily removed from the top of the film. However, once the solubility limit of N^{3-} in PC is reached, ion transport across the film becomes faster than ion transfer from the film to the liquid. This leads to a diffusion of N^{3-} from the bottom towards the top of the film, increasing the thickness of the magnetic layer generated at the bottom while neutralizing the magnetic layer at the top. Note that in (4) no magnetic field is applied, therefore, the magnetic layer being generated at the bottom becomes exchange-coupled to the pre-existing magnetic sublayer.

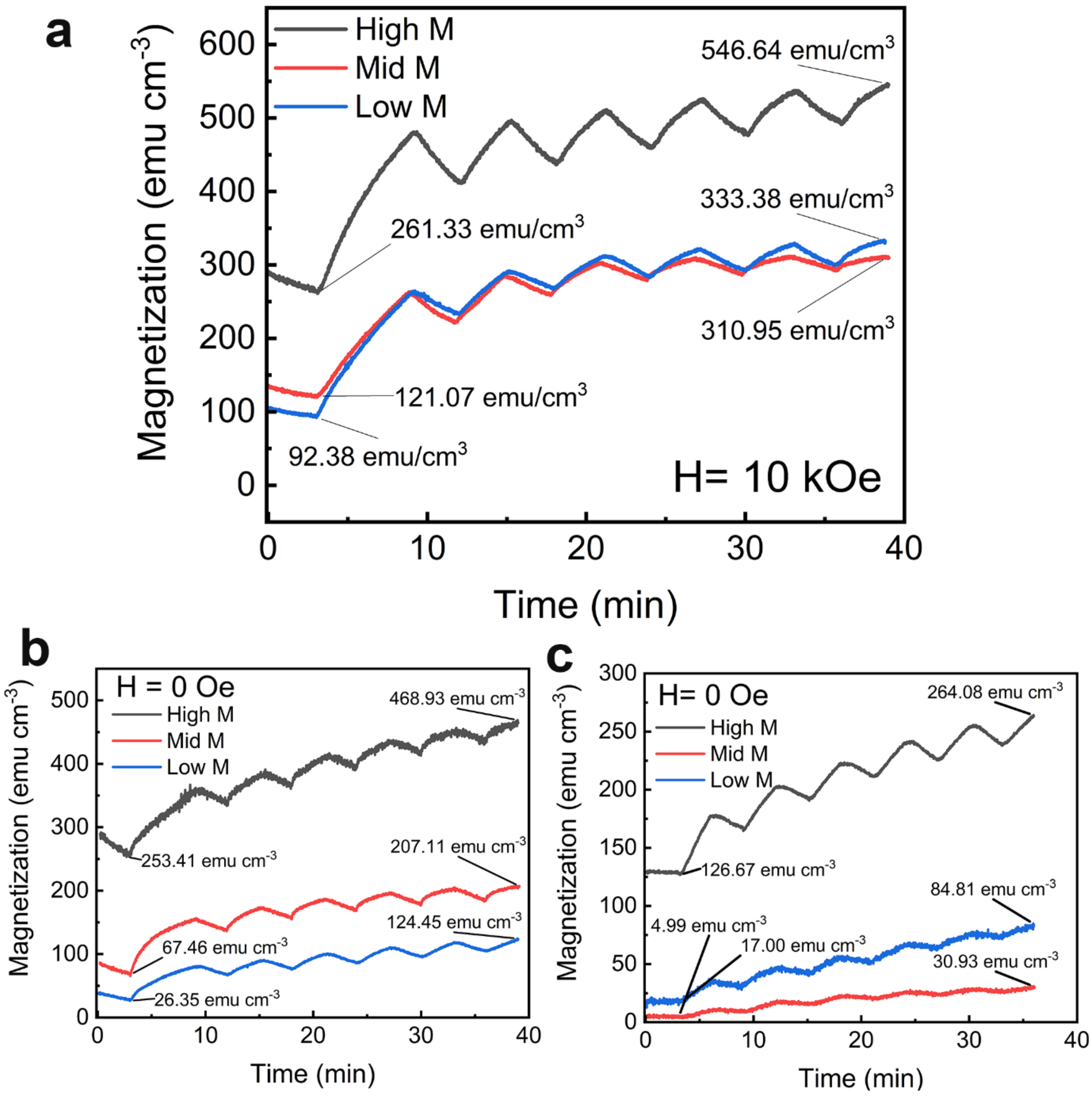

Figure S4. Magnetization vs. time plots during the different cycling experiments. a) Saturation magnetization (under H = 10 kOe) after 3 min relaxation. b) Remanent magnetization (under H = 0 Oe) after 3 min relaxation. c) Remanent magnetization (under H = 0 Oe) after 1 h relaxation.

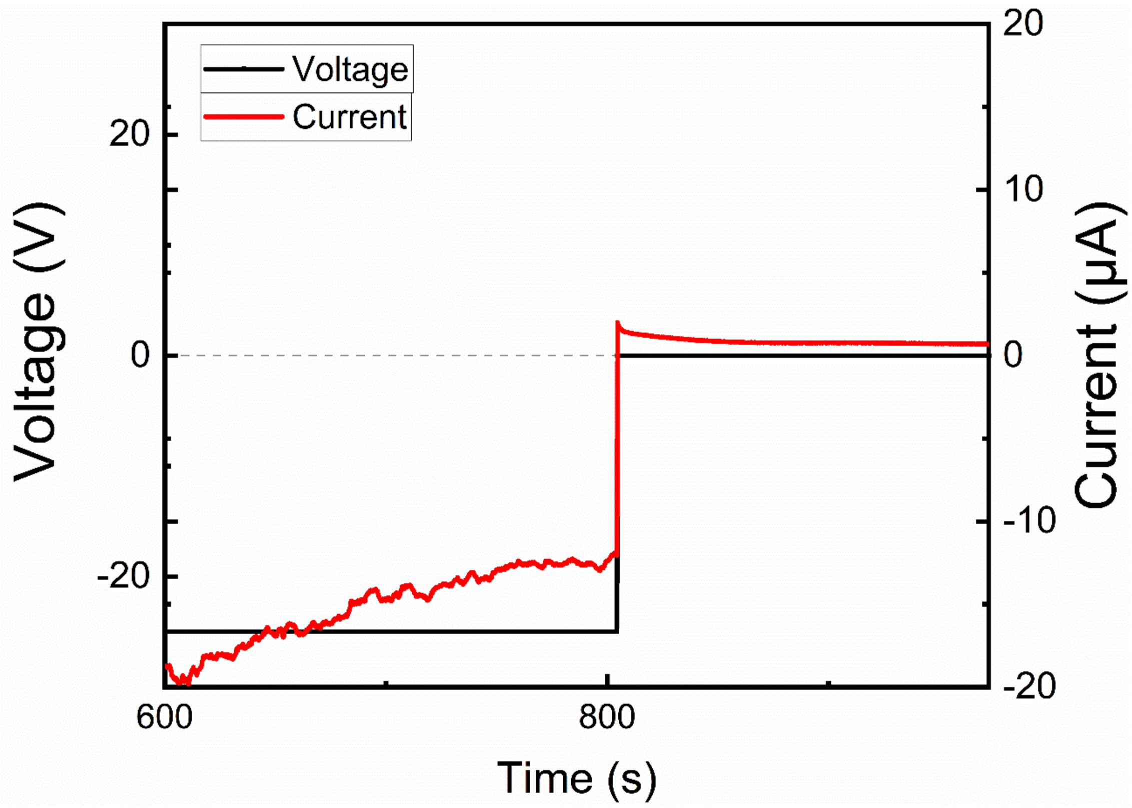

Figure S5. Applied voltage (black line) and measured current (in red) vs time.
